# Modelling ion channels with a view towards identifiability

**DOI:** 10.1007/s11538-025-01558-3

**Published:** 2025-12-09

**Authors:** Ivo Siekmann

**Affiliations:** School of Computer Science and Mathematics (CSM), Liverpool John Moores University (LJMU), Byrom Way, Liverpool, L3 3AF Merseyside United Kingdom

**Keywords:** Non-identifiability, Aggregated Markov model, Ion channel

## Abstract

Aggregated Markov models provide a flexible framework for stochastic dynamics that develops on multiple timescales. For example, Markov models for ion channels often consist of multiple open and closed state to account for “slow” and “fast” openings and closings of the channel. The approach is a popular tool in the construction of mechanistic models of ion channels—instead of viewing model states as generators of sojourn times of a certain characteristic length, each individual model state is interpreted as a representation of a distinct biophysical state. We will review the properties of aggregated Markov models and discuss the implications for mechanistic modelling. First, we show how the aggregated Markov models with a given number of states can be calculated using Pólya enumeration. However, models with  open and  closed states that exceed the maximum number  of parameters are non-identifiable. We will present two derivations of this classical result and investigate non-identifiability further via a detailed analysis of the non-identifiable fully connected three-state model. Finally, we will discuss the implications of non-identifiability for mechanistic modelling of ion channels. We will argue that instead of designing models based on assumed transitions between distinct biophysical states which are modulated by ligand binding, it is preferable to build models based on additional sources of data that give more direct insight into the dynamics of conformational changes.

## Introduction

Ion channels belong to the simplest components of living cells—in membranes that are usually impermeable to ions they open tiny pores which allow ions to enter or leave the cell. Many channels select which ions can pass through the pore but otherwise, ion channels exert little influence on the net flux of ions which is determined by a combination of the concentration gradient and the membrane potential i.e. the electrochemical gradient. Thus, at the most basic level, ion channels can be viewed as switches which are able to switch on or off fluxes of ions that would otherwise be blocked by the cell membrane.

Despite, or maybe due to their simplicity which enables them to be flexibly integrated in various physiological systems, ion channels are involved in more or less all important physiological processes. These can be roughly summarised in three categories:

1. Regulating the intracellular concentration of a particular ion.
2. Regulating the charge within a cell i.e. the membrane potential.
3. Signal transduction.

Ion channels belong to a class of cellular building blocks called membrane proteins. Although the function carried out by an ion channel can be described as one of a simple switch, the membrane protein which carries out this task is not at all simple at a biophysical level—to open a pore in the membrane, the channel protein needs to undergo one or more rearrangements of its complex three-dimensional structure—so-called conformational changes.

A fundamental question in ion channel modelling is

“Should the *biophysics of the channel protein* be reflected in a mathematical model describing the *dynamics of the ion channel*?”

We initially disregard this important question but will return to consider it after our detailed investigation of non-identifiability, see the Discussion, sec. 4.

**Fig. 1:**
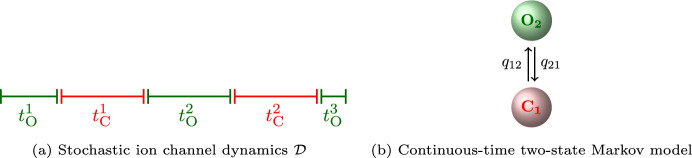
(a) The dynamics $\mathcal {D}$ of an ion channel is an alternating sequence of stochastic open and closed times  and . (b) Such a sequence can be described by a continuous-time Markov model with one closed state  and one open state . If the ion channel generates open or closed times of multiple characteristic lengths, additional open or closed states need to be added.

We describe the dynamics $\mathcal {D}$ of an ion channel as an alternating sequence of open and closed times,  (Without loss of generality we assume that the first time observed is an open time but we could equally have started with a closed time). The times  are stochastic—when an ion channel opens, it is, in principle, not possible to predict, how long it will stay open, see Fig.1.

A simple model for the alternating sequence of open and closed times $\mathcal {D}$ is the two-state continuous-time Markov model which describes stochastic transitions between a closed state  and an open state . A direct consequence of assuming that the dynamics $\mathcal {D}$ can be described by a Markov model is that the open times  and the closed times  are exponentially-distributed. Indeed, the Markov property requires that if the model has been in a state *S* for a time $t_0$, the probability that it stays in this state for an additional duration $\tau$ is independent from the time $t_0$ that has already passed. More formally,

1 $$\begin{aligned} \mathbb {P}(T> \tau + t_0) = \mathbb {P}(T>t_0) \mathbb {P}(T>\tau ), \quad t_0 \ge 0, \tau \ge 0 \end{aligned}$$

or, equivalently,2The exponential distribution is the only continuous-time distribution for which the “lack of memory” property (2) holds. A two-state Markov model can therefore just generate an alternating sequence of exponentially-distributed sequence of open and closed times. But exponentially-distributed open and closed time distributions,  and , are insufficient for describing the dynamics $\mathcal {D}$ of many ion channels—a simple counter-example is an ion channel that sometimes stays closed for a short time and sometimes for a considerably longer time. The most common approach for describing multiple timescales in the open and closed time distributions is to introduce multiple open and closed states. Intuitively, one might expect that each closed state (or open state) represents a different exponentially distributed timescale and each of these closed (or open) timescales is observed with a specific probability. Indeed, as calculating the sojourn-time distributions from a Markov model (with an arbitrary number of closed and open states) shows, this expectation turns out to be true—open and closed time distributions are mixture distributions of as many exponential components as there are open/closed states, respectively (Colquhoun and Hawkes 1981). Although the exponentials in the mixture distributions  and  can, in general, not be identified with individual states in the Markov model, it is tempting to interpret the set of open and closed states as a collection of different timescales, ranging from “slow” to “fast”.

Another attractive property of a Markov model consisting of multiple open and closed states (a so-called *aggregated Markov model* with two classes) is that one might associate individual states of the model with different biophysical states of the channel protein—one well-known example is the de Young - Keizer model of the inositol-trisphosphate receptor (IP$_3$R) (De Young and Keizer 1992). To give a simple example for this approach, consider a model that consists of a chain of multiple closed states that eventually transition to an open state, this is interpreted as transitions between various closed conformations which eventually lead to an open state i.e. a conformation in which the channel is open.

It is no coincidence that this idea has been highly influential—it suggests that a model consisting of multiple open and closed states can represent more than “just” the opening and closing of an ion channel but, in addition, can reveal the hidden dynamics of conformational changes underlying these dynamics $\mathcal {D}$. For a given number of open and closed states,  and , there are many different ways that these states can be connected to an aggregated Markov model, see Section 2 for an explicit calculation, the results are summarised in Table 1. Intuitively, it seems plausible that for a given number of open and closed states, any of the different models represent different dynamics $\mathcal {D}$. A review of long-known results on aggregated Markov models shows that this intuition turns out to be clearly wrong, as we will explain in Section 3—models with  open and  closed states can be reparametrised without altering the dynamics $\mathcal {D}$. For example, the fully-connected model for any number of states  can be continuously reparametrised so that an infinite combination of parameters can represent the same dynamics.

The phenomenon that a given model *Q* can be reparametrised to a different model $Q'$ that represents the dynamics $\mathcal {D}$ equally well is known as non-identifiability. We will consider two different aspects of this question:

1. For a given Markov model $Q=(q_{ij})$ with  open and  closed states, can the rate constants $q_{ij}$ be uniquely identified from the dynamics $\mathcal {D}$? If this is the case, the model *Q* is referred to as *parameter-identifiable*.
2. We now consider the *model structure* i.e. the graph $\mathcal {G}$ defined by the positive rates of an infinitesimal generator $Q=(q_{ij})$. We ask the question if *Q* can be reparametrised to another model $Q'$ so that they generate the same dynamics $\mathcal {D}$ with a model structure $\mathcal {G}'$ that is not equivalent to $\mathcal {G}$ as a graph (by symmetries as described in Section 2). We call two models with generators *Q* and $Q'$ *equivalent* if they produce the same dynamics $\mathcal {D}$ despite being defined on different graphs $\mathcal {G}$ and $\mathcal {G}'$. If the dynamics $\mathcal {D}$ can be generated by models defined on graphs $\mathcal {G}$ and $\mathcal {G}'$ that are not equivalent we refer to this phenomenon as *non-identifiability of model structure*.

Since the seminal papers by Fredkin et al. (1985); Fredkin and Rice (1986) it has been known that models exceeding  rate constants can, in principle, be reparametrised as in 1. i.e. these models lack parameter identifiability. We provide an outline of the derivation of this result by Fredkin et al. (1985); Fredkin and Rice (1986) in Section 3.1 as well as an alternative derivation in Section 3.2. As a consequence, we might restrict ourselves to models with fewer rate constants than this upper bound . But even models with rate constants below this bound can often be re-parametrised to models that have the same number of rate constants but whose states are connected in a different way (these models lack identifiability of model structure as in 2.)—explicit examples are presented in detail in Section 3.4. This means that even in the best case that models cannot be continuously reparametrised, there still is an equivalence class of a finite number of models that represents the same dynamics $\mathcal {D}$. This makes it challenging to interpret aggregated Markov models as representations of a particular biophysical mechanism—simply, because via reparametrisation, the dynamics of a given model can be generated by a different model that suggests a completely different mechanism.

We will then explore the implications of this non-identifiability of model structure on models that are based on representations of transitions between conformational states regulated by ligand binding sites in Section 3.5.

In the light of these difficulties, one might ask the question if aggregated Markov models are a suitable structure for representing the underlying biophysical mechanism of ion channels rather than just the dynamics $\mathcal {D}$. We have argued in Siekmann et al. (2019) that representing biophysical mechanisms such as conformational changes is unlikely to succeed by parametrising a Markov model from single channel data obtained at steady-state ligand conditions alone. Instead, it is more promising to obtain more direct evidence on the biophysical dynamics by statistically analysing modal gating (Ionescu et al. 2007; Siekmann et al. 2014) which is known to be closely linked with conformational changes or using additional data such as the response of the channel to rapid changes of ligand concentrations (Mak et al. 2007). In the Discussion (Section 4) we will explain how hierarchical Markov models Siekmann et al. (2016) and Markov models with distributed delay (Hawker et al. 2025) can be used for representing aspects of the underlying biophysical dynamics of the channel protein by providing structures that can be parametrised directly with these additional data sources.

## Enumeration of ion channel models

**Fig. 2:**
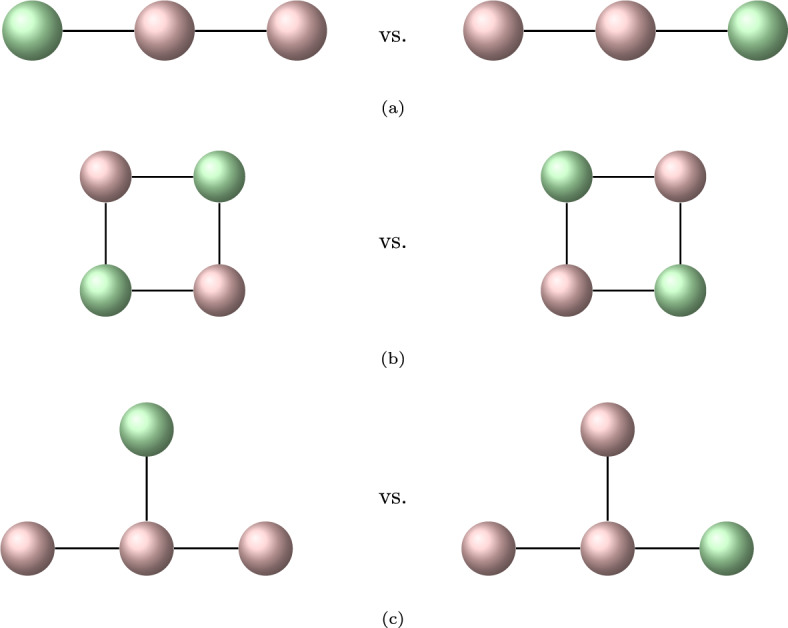
Three examples of graphs with vertices in two different colours which are equivalent (arguably, with increasing difficulty from (a) to (c)). “Equivalent” means that both are examples of the same unlabelled graph which has just been “drawn” in different ways. More formally, each graph on the left can be mapped to the graph on the right by swapping vertices without changing the adjacency structure. For a given graph which vertices can be mapped to each other is determined by the symmetry group of the graph. Enumeration of graphs with given properties relies on being able to find the symmetry group of this class of graphs and calculating the so-called cycle index, a polynomial which allows to keep track of the different equivalence classes under the operation of the symmetry group.

In Bruno et al. (2005), the number of graphs with  open and  closed states has been stated (and credited to David Torney) but further information about the derivation of these numbers have not been given. Here, the calculation of the number of graphs is explained. To illustrate the challenge we show a few examples of graphs that are equivalent, although this is not obvious at first glance, see Figure 2.

### Pólya enumeration

We will “count” the number of different graphs using a technique called *Pólya enumeration*^1^. Behind Pólya enumeration is the idea that the number of objects with a particular property can be represented by polynomials *p*(*t*).

Let us first look at the very simple example of *k*-subsets of a set of *n* elements. If 1 stands for the empty set $\emptyset$ and *t* for the set $\mathcal {S}_1 = \{1\}$ with one element, the polynomial

3 $$\begin{aligned} 1 + t \end{aligned}$$

represents the two possible subsets with zero and one element. One interpretation of the polynomial $1+t$ is that we can either draw the (single) element 1 or not.

For the set $\mathcal {S}_2=\{ 1, 2 \}$ we can extend this idea by considering the product

4 $$\begin{aligned} \underbrace{(1+t)}_{\text {draw 1?}} \cdot \underbrace{(1+t)}_{\text {draw 2?}} \end{aligned}$$

where the first factor $(1+t)$ stands for drawing the element 1 (or not) and the second factor stands for drawing element 2 (or not). It is clear that the polynomial

$$\begin{aligned} 1 \cdot 1 + 1 \cdot t + t \cdot 1 + t \cdot t = 1 + 2 t + t^2 \end{aligned}$$

enumerates all possible outcomes of drawing (without replacement) from $\mathcal {S}_2$. After simplifying the polynomial, the term 1 stands again for the empty set $\emptyset$, 2*t* represents the subsets $\{1\}$ and $\{2\}$ with one element each and $t^2$ accounts for the full set $\mathcal {S}_2=\{1, 2 \}$ with two elements. Going forward with this idea, for the set $\mathcal {S}_n=\{ 1, \dots , n\}$ with *n* elements, the process of drawing (or not) each of the *n* elements of the set can be represented via the polynomial

5 $$\begin{aligned} (1 +t)^n = \underbrace{(1+t)}_{\text {draw 1?}} \cdot \underbrace{(1+t)}_{\text {draw 2?}} \cdots \underbrace{(1+t)}_{\text {draw { n}?}} \end{aligned}$$

and via binomial expansion of (5) we obtain

6 $$\begin{aligned} (1 +t)^n = \sum _{k=0}^n \begin{pmatrix} n\\ k \end{pmatrix} t^k \end{aligned}$$

i.e. indeed the coefficient $$ \begin{pmatrix} n \\ k \end{pmatrix}$$ of each term $t^k$ indicates the number of *k*-subsets in a set with *n* elements.

In summary, the laws of polynomial arithmetic ensure that all possibilities of drawing *k* elements from a set with *n* elements are summarised appropriately.

### Enumerating graphs

**Fig. 3:**
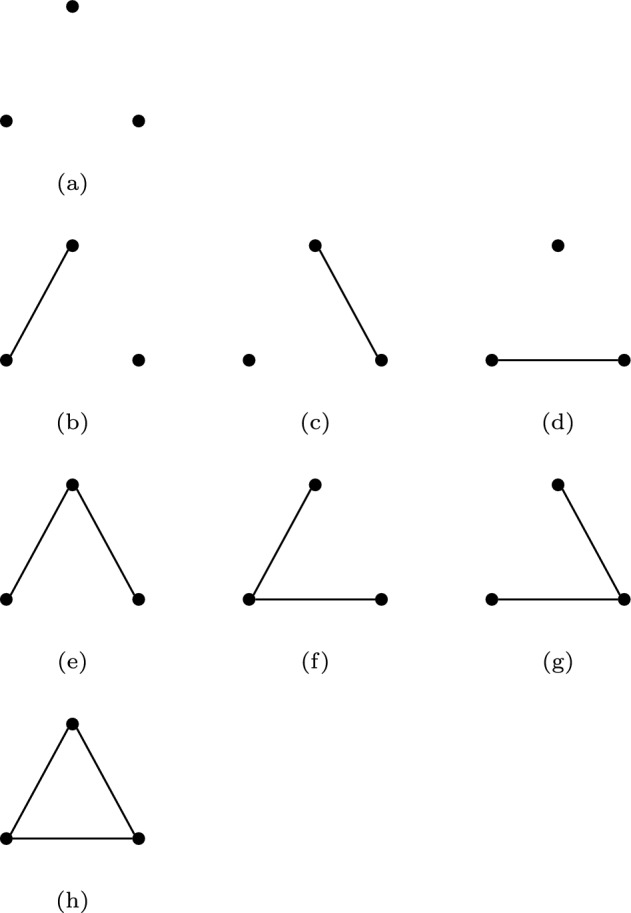
Graphs with three vertices. It is easy to see that, unless the graph is labelled, in each row, all graphs are equivalent to the first one.

We now apply the idea explained in the previous section to a method for calculating the number of unlabelled graphs for a given number of vertices^2^. A graph $\mathcal {G}$ with $n_{\text {V}}$ vertices has a maximal number of $$n_{\text {E}}= \begin{pmatrix} n_{\text {V}}\\ 2 \end{pmatrix}$$ edges. Similar to the calculation of the *k*-subsets of the set $\mathcal {S}_n$ with *n* elements, the idea of enumerating graphs is based on drawing without replacement from the edge set. Unfortunately, the sequence of coefficients $$ \begin{pmatrix} n_{\text {E}}\\ k \end{pmatrix}$$ of the polynomial $(1+t)^{n_{\text {E}}}$ does not yield correct results for graphs with more than two vertices. For example, for a graph with $n_{\text {V}}=3$ vertices (which has up to $n_{\text {E}}=3$ edges), the formula yields $1 + 3 t + 3 t^2 + t^3$, indicating that there are 3 graphs with one edge and 3 graphs with two edges. This is the correct result for a labelled graph i.e. a graph where any pair of vertices (and thus, also edges between them) can be distinguished from each other. If vertices are not labelled, any rearrangement of vertices that leaves the edge structure of the graph unchanged, is considered to represent the same graph, see Fig. 3.

#### Symmetry group of a graph—the line group

**Fig. 4:**
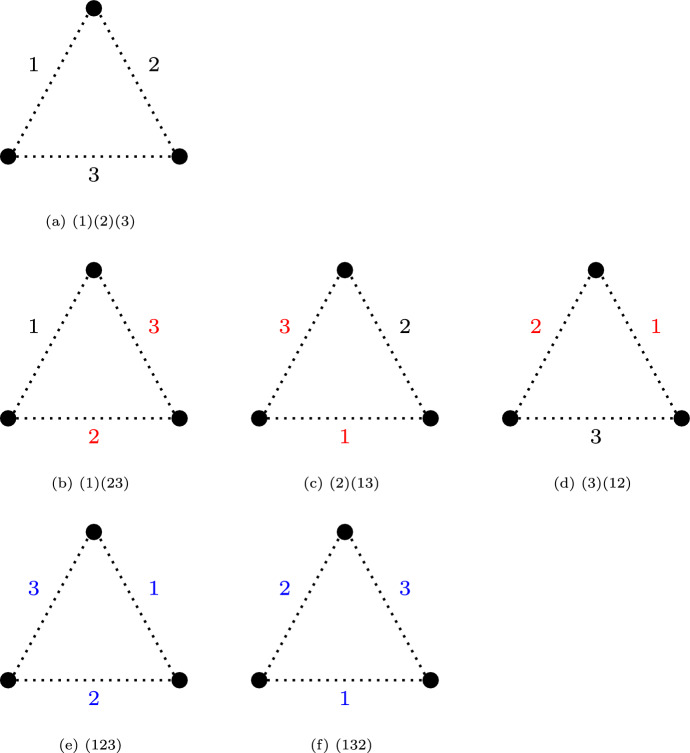
Representation of the six different permutations of the graph with three vertices.

Because we enumerate graphs by the number $n_{\text {E}}$ of edges we consider the symmetry group on the edge set of the graph, the line group. As an example, the symmetries of a graph with $n_{\text {V}}=3$ vertices are shown in Fig. 4.

Each symmetry is a permutation of the edges of the graph and we will represent each permutation via disjoint cycles. Within a cycle $(\cdots )$, each element is mapped to the subsequent element and the last element is mapped to the first one that appears in the cycle (hence the name “cycle”). For example, the cycle (132) maps edge 1 to edge 3, edge 3 to edge 2 and edge 2 to edge 1, see Fig. 4f. The cycle (23) swaps edges 2 and 3, in order to account for what happens to edge 1, we need to add the cycle (1) to account for the fact that edge 1 is left unchanged so that we obtain the cycle representation (1)(23) for the permutation shown in Fig. 4b. It is well-known that any permutation can be represented via *disjoint cycles* i.e. each element appears in any of the cycles exactly once. For a given cycle $(\cdots )$, the number of elements that appear within the cycle are the length of the cycle. For example, the permutation (1) (23) consists of one cycle of length 1 and one cycle of length 2.

#### The cycle index

The disjoint cycle representation is important because it will help us construct a multivariate polynomial, known as the *cycle index* which will enable us to account for the effect of permutations on the number of elements in a set after identifying equivalent elements. The cycle index is usually introduced in a very abstract form which makes it applicable to a wide range of applications but makes it difficult for those unfamiliar with the general theory to understand how it is applied in a particular situation. For our purpose of understanding the specific example of enumerating graphs we hope that the reader will find it simpler to consider the concrete example of a graph with three vertices to understand the principle of the construction of the cycle index. A general presentation of the theory can be found in Pólya (1937); Harary and Palmer (1973).

The cycle index represents each permutation that has been written in the disjoint cycle representation via a multivariate monomial $t_1^{\alpha _1} t_2^{\alpha _2} \dots \ t_n^{\alpha _n}$. Here, each variable $t_i$ stands for a cycle of length *i* and the exponent $\alpha _i$ accounts for how many times a cycle of length *i* occurs in a given permutation. This means that, for example, the permutation (1)(2)(3) i.e. the identity is represented as $t_1^3$, for (1)(23) we obtain $t_1 t_2$ and for (132) we have $t_3^1$.

The benefit of introducing the variables $t_i$ is that they allow us to account for the effect of a cycle of length *i*. Consider, for example, the permutation (1)(23), see Fig. 4b. Because edges 2 or 3 are swapped, the resulting graph can only be equivalent if both edges are either present or absent i.e. for a cycle of length 2 we need to either draw none of the edges in the circle or both. This can be represented by the polynomial $1+t^2$ where $t^2$ represents drawing two edges. Because edge 1 is not moved we can either include this edge in the graph or not which is represented, as above, as the polynomial $1+t$. In general, to see the effect of a cycle of length *i* we replace the variable $t_i$ with $1+t^i$.

To account for multiple permutations with a particular combination of cycles—these permutations are said to have the same type—we multiply each monomial with the number of permutations of the type represented by this monomial.

The cycle index of the line group $\mathfrak {S}^{(2)}_3$ of graphs with $n_{\text {V}}=3$ vertices is

7 $$\begin{aligned} Z(\mathfrak {S}^{(2)}_3; t_1, t_2, t_3) = \frac{1}{6} \left( t_1^3 + 3 t_1 t_2 + 2 t_3 \right) \end{aligned}$$

where $t_1^3$ accounts for the identity, $3 t_1 t_2$ stands for the 3 permutations that swap two edges while not moving the remaining one and $2 t_3$ represents the two permutations that move all three edges, see Fig. 4. The polynomial is scaled by the number of permutations in the symmetry group which results in the factor $\frac{1}{6}$.

According to Pólya’s enumeration theorem, by evaluating the cycle index $Z(\mathfrak {S}^{(2)}_3, 1+t, 1+t^2, 1+t^3)$ we can find a polynomial that represents the number of graphs with 3 vertices for different numbers of edges:

8 $$\begin{aligned} Z(1+t, 1+t^2, 1+t^3)&= \frac{1}{6} \left[ (1+t)^3 + 3\cdot (1+t)(1+t^2) + 2\cdot (1+t^3) \right] \end{aligned}$$

9 $$\begin{aligned}&= 1 + t + t^2 +t ^3. \end{aligned}$$

This result indicates that among graphs with three vertices we can find one unlabelled graph each with zero, 1, 2 or 3 edges. This is consistent with Fig. 3.

Based on this example, we can conclude that Pólya enumeration relies on finding an expression of the cycle index for the symmetry group for a particular class of objects of interest. This is not an easy task and leads to a quite complicated closed-form formula in the general case. Because the main aim of this section is to explain the underlying idea of the enumeration of aggregated Markov models rather than explaining the calculation of the line group $\mathfrak {S}^{(2)}_n$in full detail we refer the reader to chapter 4.1 in Harary and Palmer (1973). The number of graphs for a given number of vertices $n_{\text {V}}$, calculated in this way can be found in Table 1.

### Graphs with coloured vertices and rooted graphs

**Fig. 5:**
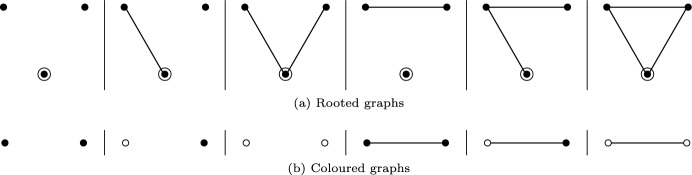
Rooted graphs with 3 vertices and the corresponding coloured graphs with 2 vertices. Vertices that are adjacent to the root are represented as white in the corresponding coloured graph, vertices that are not adjacent are black.

Enumerating aggregated Markov models comes with the additional difficulty that the vertices of the graph can have two different “colours”, say red and green, as in Fig. 3. An elegant solution for enumerating graphs with two colours is to consider rooted graphs, see Figure 5.

“Colour” is represented in a rooted graph by considering if a designated root node is adjacent to a vertex or not—in Fig. 5, a vertex which is adjacent to the root is coloured white, if it is not adjacent to the root it is coloured black. Representing the colour of a vertex as part of the structure of the graph is much simpler than attempting to investigate the different colourings of graphs under their respective symmetry groups. The calculation of the cycle index for rooted graphs is again described in Harary and Palmer (1973, chapter 4.4), results for the number of rooted graphs for a given number of vertices $n_{\text {V}}$ are found in Table 1.

### Connected rooted graphs... and The End

In our attempt to enumerate the number of aggregated Markov models there is one difficulty that remains. Instead of all graphs whose vertices can have two colours we are, in fact, only interested in connected graphs. There is a general method for determining the number of connected graphs from the number of graphs with a given property. For this, we consider the formal power series (also known as the generating series)

10 $$\begin{aligned} g(t) = \sum _{k=1}^{\infty } g_k t^k \end{aligned}$$

where $g_p$ is the number of graphs with a certain property, for example, the number of arbitrary graphs or the number of rooted graphs with *k* vertices—note that in contrast to the examples for enumeration of graphs and rooted graphs considered above we are not enumerating graphs by the number of edges.

If we define, analogously to (10), the generating series *c*(*t*) of connected graphs or rooted graphs, *g*(*t*) and *c*(*t*) are related by *Riddell’s formula*^3^ (Riddell 1951; Harary and Palmer 1973):

11 $$\begin{aligned} 1 + g(t) = \exp \left( \sum _{k=1}^\infty \frac{1}{k} c(t^k) \right) . \end{aligned}$$

This relationship between graphs and connected graphs might look rather mysterious but it is based on the simple fact that for any graph we can determine the number of connected components. Thus, the number of graphs can be reconstructed by drawing connected graphs that will then become the connected components of a regular graph. The additional term $+1$ in (11) stands for the “empty” graph with no vertices. The relevant symmetry group for graphs consisting of *n* connected components is $\mathfrak {S}_n$ so that we obtain the cycle index

12 $$\begin{aligned} \sum _{k=1}^{\infty } Z(\mathfrak {S}_n; c(t)) =: Z(S_\infty , c(t)). \end{aligned}$$

It can be shown that as a formal power series,

13 $$\begin{aligned} Z(\mathfrak {S}_\infty ; c(t)) = \exp \left( \sum _{k=1}^\infty \frac{1}{k} c(t^k) \right) . \end{aligned}$$

By taking advantage of the arithmetic of formal power series—although this involves some tedious calculations—it is possible to use (11) for calculating the generating series *c*(*t*) for connected graphs from the generating series *g*(*t*) for arbitrary graphs. This process is also known as an inverse Euler transform of (11).

One possible solution is to set

14 $$\begin{aligned} \sum _{k=1}^\infty a_k t^k = \log ( 1 + g(t)) \end{aligned}$$

and calculate the coefficients $a_k$ by coefficient matching with $\log ( 1 + g(t))$ as a power series. This yields the recursion formula

15 $$\begin{aligned} a_k = g_k - \frac{1}{k} \sum _{i=1}^{k-1} i \cdot a_i g_{k-i}. \end{aligned}$$

Matching coefficients of $\sum _{k=1}^\infty a_k t^k$ with $\sum _{k=1}^\infty \frac{1}{k} \cdot c(t^k)$ leads to the relationship

16 $$\begin{aligned} a_k = \frac{1}{k} \sum _{d | k} d c_d. \end{aligned}$$

Finally, the coefficients $c_k$ of the generating series for connected graphs can be calculated from $a_k$ by *Möbius inversion*:

17 $$\begin{aligned} c_k = \sum _{d | k} \frac{\mu (d)}{d} a_{p/d} \end{aligned}$$

where *d*|*k* stands for all divisors *d* of *k* and the Möbius function is defined as

18 $$\begin{aligned} \mu (k) = \left\{ \begin{array}{ll} 1 & \text { if } n=1,\\ (-1)^k & \text { if } n \text { is a product of } k \text { distinct primes},\\ 0 & \text { if } n \text { is divisible by a square}. \end{array} \right. \end{aligned}$$

Using the formulae (15) and (17) it is straightforward to calculate the number of connected graphs as well as the number of rooted graphs, see Table 1.

The number of connected rooted graphs with $n_{\text {V}}+1$ vertices (including the root node) or, analogously, graphs with $n_{\text {V}}$ vertices which have one of two different colours, also contains graphs that have only one of the two colours. Those graphs are examples for degenerate aggregated Markov models where only one of the two aggregates is present. Fortunately, it is not difficult to exclude these graphs—for every connected graph there are only two graphs that have only one colour—one in each colour. Thus, the number of (non-degenerate) aggregated Markov models $M_k$ with $n_{\text {V}}$ vertices is obtained from the number of coloured graphs $CR_k$ by subtracting twice the number of connected graphs $2 c_k$:

19 $$\begin{aligned} M_k = CR_k - 2\cdot c_k. \end{aligned}$$

**Table 1:** Enumeration of graphs, rooted graphs, connected rooted graphs and aggregated Markov models obtained by Pólya enumeration as explained in Section 2. Note that for rooted and connected rooted graphs we have not counted the root node i.e. any rooted graphs with $n_{\text {V}}$ vertices has $n_{\text {V}}+1$ vertices including the root.

$n_{\text {V}}$	Graphs	Connected graphs	Rooted graphs	Connected rooted graphs	Aggregated Markov models
1	1	1	2	2	0
2	2	1	6	3	1
3	4	2	20	10	6
4	11	6	90	50	38
5	34	21	544	354	312
6	156	112	5096	3883	3659
7	1044	853	79264	67994	66288
8	12346	11117	2208612	2038236	2016002
9	274668	261080	113743760	109141344	108619184
10	12005168	11716571	10926227136	10693855251	10670422109

## Non-identifiability

One aspect of aggregated Markov models is that non-identifiability is a common phenomenon. Non-identifiability can be illustrated by the hypothetical situation that we know exactly the structure of the underlying model that generates the process that we observe. If the model is non-identifiable, it can generate identical behaviour when choosing different parameter sets. The reason why such a model is called non-identifiable can be explained by the somewhat counter-intuitive consequence that even if an infinite amount of data which is not perturbed by noise were available, the parameters of the model nevertheless could not be uniquely determined by fitting the model to these data.

### Upper bound for the number of parameters in aggregated Markov models

The main result in this regard goes back to Fredkin et al. (1985); Fredkin and Rice (1986) who observed that the dynamics of aggregated Markov models can be completely represented by the bivariate distributions  and  of the length of an open time  followed by a closed time  and vice versa :20212223Note that here, the parameters  and  are the negatives of eigenvalues of submatrices of the infinitesimal generator *Q* of the aggregated Markov model.

The bivariate distributions (20), (22) can also be represented as bilinear forms. By defining the matrices2425we obtain2627Using the result from (Fredkin et al. 1985; Fredkin and Rice 1986) that all information on the dynamics of an aggregated Markov model is contained in the bivariate distributions  and  (Theorem 4.1 in Fredkin et al. (1985), and Theorem A in Fredkin and Rice (1986))^4^, we can find the maximal number of free parameters of an aggregated Markov by answering the simpler question how many parameters the distributions  and  depend on. The numbers of free parameters in the univariate distributions  and  are contained in this number because these distributions can be obtained by marginalising the bivariate distributions, for example,2829To determine the number of parameters contained in the distributions  and 5 we follow the derivation given in Fredkin et al. (1985, Corollary 4.1).

To find the total number of free parameters contained in  and  we consider (22) or (26) and observe that we have to account for the  rates  and the  rates  and add them to the number of parameters contained in  and . Considering that the  and  can be interpreted as tables with  parameters each, the number of parameters that both  and  depend on appears to be  so that the total number of parameters would be . But there are a few constraints that reduce this number.

First of all, considering only one of the distributions, say , we observe that the number of parameters is reduced by one—because  is a probability distribution, the  , see (21), have to sum up to one. This yields  free parameters.

Second, there are constraints that arise from the coupling of  and  introduced by the fact that  and  can both be obtained from the bivariate distributions  and  via marginalisation. Calculating  by marginalising  as shown in (28) is just one of two possibilities— can also be calculated analogously to (28) from the distribution . Whilst marginalising  yields coefficients , marginalising  leads to coefficients  that depend on the  instead. Because calculating the coefficients  from  instead of  must not change their value, we obtain the constraints30and analogously, by considering the calculation of  via marginalisation of  and , respectively, further constraints31on the  are obtained. Interpreting  as a  matrix, these constraints allow us to calculate one element in each column from the equations in (30) and, similarly, using (31), one element in each row of  from the  and other entries in the same row or column. This means that the  matrix   effectively has only  components. Thus, in total counting the number of independent parameters contained in the coefficients  and  we conclude that there are32Now, taking into account that there are  rate constants  and  rate constants  we find that in total there are at most  independent parameters in the distributions  and .

We note that a refined upper bound for the maximum number of rates of an aggregated Markov model has been derived in Fredkin and Rice (1986). Apart from the numbers of open and closed, Fredkin and Rice (1986) also considered the rank of the transmission matrices  and . For more details we refer the reader to Lemma A in Fredkin and Rice (1986) where this result is presented.

### Alternative derivation of the upper bound 

We would like to derive the upper bound  for the number of rate constants of an aggregated Markov model more directly, as presented previously in similar form in Kienker (1989). For this purpose it is useful to write the infinitesimal generator *Q* of an aggregated Markov in the form33Although the matrix $\textbf{Q}$ has  components, for the diagonal elements $q_{ii}$ we have $q_{ii} = - \sum _{j\ne i} q_{ij}$. Thus, at most  components of $\textbf{Q}$ can be freely chosen—this coincides with the number of directed edges in a complete graph with  vertices and therefore the maximal number of rates.

But if the blocks  and  are diagonalisable it is possible to reparametrise the model $\textbf{Q}$ so that all components within  and  except for the diagonal vanish—without changing the dynamics $\mathcal {D}$! Thus, we consider block-wise similarity transformations $\textbf{S}$ introduced by Kienker (1989)34where  and  are invertible matrices and ,  are matrices of zeroes. It is easy to see that35If we consider models $\textbf{Q}'=\textbf{S}^{-1} \textbf{Q} \textbf{S}$, it is well-known that the eigenvalues  and  are invariants but Kienker (1989) demonstrates in his Lemmata 1 and 2 that even the probability distributions , ,  and  are preserved under block-wise similarity transformations. This means that the dynamics $\mathcal {D}$ of a model $\textbf{Q}'=\textbf{S}^{-1} \textbf{Q} \textbf{S}$ is indistinguishable from the model $\textbf{Q}$. For this reason, the models $\textbf{Q}'$ and $\textbf{Q}$ are called *equivalent*.

The upper bound  can now be easily derived if the block matrices  and  are diagonalisable. This is true for models that fulfil the *detailed balance conditions* (57) which will be discussed in more detail below. Because the detailed balance conditions are related to the thermodynamic principle also known as the Second Law of Thermodynamics that entropy can never decrease in a closed system (when interpreting a Markov model as a representation of a chemical system), detailed balance is often assumed for Markov models of ion channels.

For diagonalisable  and , we can find a matrix  that brings the blocks  and  to diagonal form:3637From (37) it is now clear that the transformed matrix can at most depend on  free parameters—the diagonal elements $q'_{ii}$ can again be disregarded because they are determined by the off-diagonal elements and the matrices  and  have  parameters each.

This calculation also shows that it is possible to find a reparametrisation of any fully connected aggregated Markov model to an aggregated Markov model with  rate constants (which therefore fulfils the necessary condition for identifiability). However, as we will see in Section 3.4.1 where this transformation is carried out explicitly for the fully connected three-state model, this does not always yield a feasible model—as we will observe, there are parameter sets for which some of the rates in the reparametrised model are negative.

The matrix (37) defines a model structure where all transition rates from open to other open states or between closed states vanish—all transitions occur between open and closed states. This structure has been proposed as a *canonical from* for models with a given number of  open and  closed states. Canonical forms provide models that are parameter-identifiable and can be used as representatives for equivalence classes of models. The particular form (37) has been studied by Bauer et al. (1987); Kienker (1989) and is referred to as *Bauer-Kienker uncoupled (BKU) form* in Bruno et al. (2005). For a brief discussion of canonical forms see the Discussion (Section 4).

### Interpretation of sojourn time distributions

**Fig. 6:**
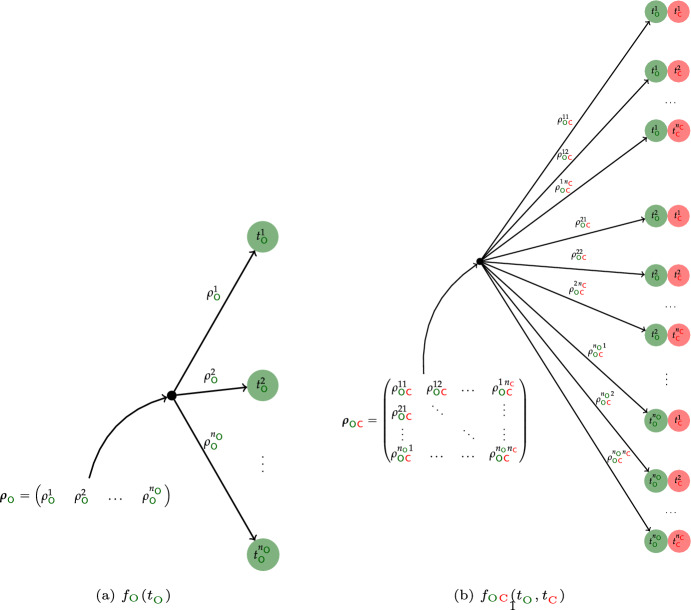
Graphical illustration of the sojourn time distribution  and the bivariate distribution  as mixtures of exponential distributions—the distributions  and  are analogous. It shows that sojourn times  are generated in a two-stage process. First, from the finite distribution defined by , an exponential distribution  is chosen which then generates an open time , see (a). Similarly, the matrix  defines a finite distribution over pairs of exponential distributions. After choosing a pair , , , it generates an open time  and a subsequent closed time , see (b).

The reduction of aggregated Markov models to the bivariate distributions  and  (Fredkin et al. 1985; Fredkin and Rice 1986) leads to an alternative interpretation of the dynamics $\mathcal {D}$. This leads to a more abstract view compared to the illustrative transitions between open and closed states which are assigned additional biophysical significance by associating them with different conformational states of the channel protein. Figure 6 illustrates that the distributions , ,  and  can be interpreted as two-stage processes where a finite distribution is used for determining which distributions are used for generating either sojourn times or pairs of subsequent sojourn times. Figure 6a illustrates how the coefficients  define a finite distribution that stochastically selects if an open time is generated from a “slow” or “fast” exponential state. As shown in Figure 6b this is extended to the distribution  which generates an open time followed by a closed time. After an open and a closed exponential state have been chosen by the finite distribution defined by the components  of the matrix , a pair  of subsequent open and closed times is generated.

### *Example:* The fully connected three-state model

In order to illustrate the consequences of this abstract result by Fredkin et al. (1985); Fredkin and Rice (1986) with a concrete example, let us consider the simplest case of a non-identifiable model, the fully connected three-state model. We follow and expand the presentation from Kienker (1989):38The matrix *Q* in (38) is partitioned in the closed compartment  and the open compartment . It is helpful to summarise the parameters of the model via the sojourn time distributions  and :3940The two distributions  and  together depend on four independent parameters—the weight $\rho$ of the first component in the mix of two exponential distributions that  consists of as well as the rates ,  and  which are eigenvalues of submatrices of the matrix *Q*.

Because eigenvalues depend in an increasingly complex way on the components of a matrix as the dimension increases, it is advantageous to alternatively consider41424344when evaluating transformations of the generator *Q*. In order to investigate non-identifiability, we are interested in reparametrisations of the matrix *Q* that alter the rate constants $q_{ij}$ but leave the parameters of the sojourn time distributions invariant.

For the three-state model, the rates of the closed-time distribution  are obtained as solutions of the characteristic polynomial of the matrix 45The weight $\rho$ can be found by elementary but relatively tedious calculations46Because, in order to leave the closed time distribution  unchanged, the parameter $\rho$ needs to be preserved by a reparametrisation and the expression (46) shows that *S* must be an invariant when reparametrising the model because all other parameters on the right-hand side of (46) are invariants. It also shows that it is possible to parameterise the three-state model, so that $\rho <0$. In this case, the closed-time distribution  is a signed mixture of exponential distributions, rather than an ordinary mixture distribution for which all coefficients must be positive.

We will see in the following Section 3.4.1 that for $\rho >0$ where  is a mixture of exponential distributions, the fully connected three-state model can be transformed to a reduced model with fewer rate constants, decreasing the original 6 parameters to a model with 4 parameters. In contrast, this will not be possible if  is a signed mixture of exponential distributions.

#### Transformation of the general three-state model to the model 

We will attempt to reparametrise the model *Q* to obtain a model $\tilde{Q}$ with the same sojourn time distributions  and . The model  is a special case of the three-state model where both closed states $C_1$ and $C_2$ are adjacent to the open state $O_3$ but direct transitions between the closed states are not possible—$\tilde{q}_{12}=\tilde{q}_{21}=0$. Because the matrix  is diagonal it follows that the rate constants $\tilde{q}_{13}$ and $\tilde{q}_{23}$ must coincide with the eigenvalues  and . For the remaining rate constants, in order to keep  and *S* invariant, we obtain linear equations in $\tilde{q}_{31}$ and $\tilde{q}_{32}$:4748Interestingly, the solution49so that the model  can be easily obtained from the distributions  and  by assigning the parameters $\rho$, ,  and  to the appropriate rate constants of the model  (this is, of course, the specific example of the transformation used in Section 3.2 to derive the upper bound  for the maximum number of rates for the model with $n_{\text {V}}$ vertices):50It seems that the non-identifiability problem—at least for the simple case of the full three-state model—has a very simple solution (deceptively simple as it turns out). Instead of considering the complete parameter space of the fully connected three-state model, the model is reduced to an equivalent  model.

But this overlooks that this reduction is not always possible in a meaningful way—for some possible parameter choices of three-state model, a negative value for $\rho$ is obtained. In this case, the rate constant $\tilde{q}_{31}$ is negative which contradicts the underlying assumption that the rate constants of a Markov generator are all non-negative.

#### Continuous reparametrisation of the fully connected three-state model

The fully connected model cannot only be transformed to the model  but, in fact, to a continuous set of models parametrised by two of the rates. By considering that the quantities in (41)-(43) need to be invariant to preserve the sojourn time distributions  and  these quantities can be taken as invariants that different parametrisations need to fulfil. We can, for example, choose $q_{13}$ and $q_{23}$ as free parameters and express the remaining rate constants as functions of $q_{13}$ and $q_{23}$ for given  and *S*.

From the relationship51we can calculate:52This expression for $q_{21}$ allows us—using the trace *Tc* in (41)— to derive53Similarly, with *S* from (42) after replacing $q_{32}$ using the trace , equation (43), we can calculate:54and finally55

#### Transformation of the general three-state model to the model 

Setting $q_{13}=0$ in the equations (52)-(55) we can calculate the rates of a model where $q_{13}$ vanishes:56Interestingly, $q_{13}=0$ does not imply $q_{31}=0$. But $q_{31}$ does vanish if we require the *detailed balance conditions*. By definition, the detailed balance conditions are575859If a vector $\boldsymbol{\pi }$ exists that fulfils these conditions it is a *stationary distribution* of the Markov model. It can be shown that these conditions are fulfilled provided that

60 $$\begin{aligned} q_{31} q_{12} q_{23}= q_{13} q_{32} q_{21}, \end{aligned}$$

in general, the products of rates along all cycles that involve three or more states must be equal in either direction. With detailed balance it, of course, follows that if $q_{13}=0$,  must vanish as well—if $q_{13}=0$ the left-hand side of (60) vanishes which implies that one of the rates on the left-hand side must be zero as well. Thus,  and we have61It is also possible to find a version of the model  for which $q_{12}=0$ but $q_{21} \ne 0$ (this model does therefore not fulfil detailed balance). Substituting  in (52) and (54) and rewriting $q_{31}$ and $q_{32}$ using the expression for $\rho$ from (46) yields62For the resulting model in (62) it shows that for $q_{12}=0$, an infinite set of equivalent models can be obtained by continuously varying .

**Fig. 7:**
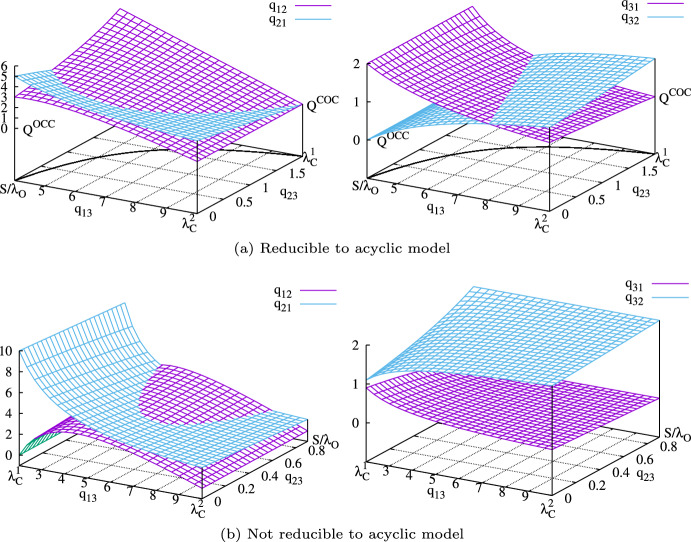
Summary of the analysis of the complete three-state model. The rates $q_{13}$ and $q_{23}$ connecting the closed states $C_1$ and $C_2$ with the open state $O_3$. Without loss of generality we assume $q_{13}>q_{23}$ and . In (a), . In this case, for all choices  and , parametrisations can be found which produce the same stochastic dynamics. The parameter sets obtained for combinations of $q_{13}$ and $q_{23}$ along the curve on the base of the plot fulfil detailed balance, see (60). For , $q_{23}=0$ we obtain the model , for  we obtain the model . The situation is fundamentally different in (b) where . Here, the fully connected three-state model cannot be reduced to the acyclic model  or  with non-negative rates. Also, in this case the closed time distribution  is not a mixture but a signed mixture of exponentials which generates a non-monotonous distribution. The set of equivalent models with non-negative rates in this case extends over the intervals  and . The detailed balance condition (60) cannot be fulfilled for any of these models.

#### Summary

Our analysis of the fully connected three-state model has illustrated the two aspects of identifiability introduced in the introduction— parameter identifiability and the identifiability of model structure.

Our analysis shows that, at least for the example of the fully connected three-state model, *parameter identifiability* and *identifiability of model structure* are closely linked as visualised in Figure 7. Both Figures 7a show parameter sets $q_{ij}$ of the fully-connected three-state model that preserve the four invariants , ,  and *S*, see (41)-(43). Because the three-state model is parametrised by six rates, we obtain an equivalence class that depends on two parameters such as, for example, the rates $q_{13}$ and $q_{23}$, see Section 3.4.2. Both rates are constrained to finite intervals in order to ensure that all six rate constants of the three-state model are non-negative.

When considering this equivalence class of models, two fundamentally different cases can be distinguished—without loss of generality, we assume that :

1. If , the models  and  are contained in the equivalence class. In this case, parameter identifiability can be resolved by selecting either  or  as a representative of the equivalence class. In this way, non-identifiable models with six rates can be *reduced* to either of the two identifiable models  or  with four rates. This case is visualised in Figure 7a.
2. If , it is not possible to reduce models in the equivalence class to either  or  so that the rates are all non-negative. Thus, in this case, the parameter non-identifiability cannot be resolved because it is not possible to choose any of the parameter-identifiable models  or  as a representative. Also, in this case, the detailed balance conditions (57) cannot be fulfilled. Parameter sets of this type are visualised in Figure 7b.

By considering (46), we find that the condition  is equivalent to $\rho >0$. This means that models are reducible to an identifiable model if the closed time distribution  is a mixture of exponentials. In the opposite case  we find, in contrast, that the closed time distribution is a signed mixture of exponential distribution—here, the coefficients $\rho$ and $1-\rho$ of the closed time distribution  sum to one but either $\rho$ or $1-\rho$ is negative.

Because in this case, where the closed time distribution  is a non-monotonous signed mixture of exponentials, it is not possible to find a representative that is parameter-identifiable, this means that there are dynamics $\mathcal {D}$ which cannot be represented by an identifiable model which means that it is not possible to identify an unambiguous “mechanism” that can be represented using aggregated Markov models which generates this dynamics $\mathcal {D}$. To the best of our knowledge this phenomenon which deserves further attention has not been systematically investigated for aggregated Markov models with a larger number of states.

### Model structure and biophysical mechanism$^{6}$

6 We will now investigate the question if natural mechanistic models for the dependency of a ligand-gated ion channel on the ligand concentration *c* can help us decide which of the models  or  might be preferable for a given data set. Although we have shown previously that both models are equivalent, they represent quite different mechanisms if we assume that the transitions between the model states are modulated by ligand binding sites. The model  represents two *independent ligand binding* sites which can either facilitate or inhibit the transition to the open state. In contrast,  models *sequential binding*—a first ligand binding site increases the transition rate from the closed state  to whereas a second binding site (which becomes accessible when the channel has transitioned to ) then activates the channel. To transform between the two models we can use (50) and (56) to directly relate the rates of the models  and :63646566One aspect of (63)-(66) is that these expressions clearly show that an arbitrary model of the from  can be transformed to a  model—if the rates , ,  and  are positive, the rates , ,  and  or , ,  and  of the transformed model are positive as well.

But our primary interest is to investigate the effect of this transformation for different assumptions on the ligand dependencies of the model . For simplicity, we will assume that all dependencies are modelled via mass action. We will drop the superscripts for the rates of the models  and , instead we will use $q_{13}, q_{23}, q_{31}$ and $q_{32}$ when we refer to rates of the model  and $\tilde{q}_{12}, \tilde{q}_{13}, \tilde{q}_{21}$ and $\tilde{q}_{31}$ when we refer to rates in the model  obtained via transformation of the model .

#### Two activating binding site

First, we assume that the channel has two activating binding sites. This implies that the rates $q_{13}$ and $q_{23}$ entering the open state are ligand-dependent and the rates $q_{31}$ and $q_{32}$ are constant i.e. independent from the ligand concentration *c*:

67 $$\begin{aligned} q_{13} = k_{13} c, q_{23} = k_{23} c, q_{31} = k_{31}, q_{32} = k_{32}. \end{aligned}$$

Replacing (63)-(66) we observe that the resulting rate constants for the model  are:

68 $$\begin{aligned} \tilde{q}_{12} = \tilde{k}_{12} c, \tilde{q}_{21} = \tilde{k}_{21} c, \tilde{q}_{23} = \tilde{k}_{23} c, \tilde{q}_{32} = \tilde{k}_{32}. \end{aligned}$$

with, as can simply be seen,69707172From a modelling point of view, the resulting  model is unusual. It appears that the activating binding sites that facilitate the transitions rate from the closed states  and  to the open state  translate to activating binding sites that enable the transition from  via  to . But although unbinding from binding sites would normally assumed to be ligand-independent, the transition $q_{21}$ from  to  is again a mass action rate dependent on *c*. This implies that in order to reproduce the behaviour of the  model, a ligand-dependent rate that inhibits the channel for large *c* by transitioning to the state  seems to be required.

#### One activating binding site

Now, we assume that, of the two transitions to the open state, one is activated by ligand binding whereas the other transition is ligand-independent:

73 $$\begin{aligned} q_{13} = k_{13} c, q_{23} = k_{23}, q_{31} = k_{31}, q_{32} = k_{32}. \end{aligned}$$

Replacing (63)-(66) we observe that the resulting rate constants for the model  are:

74 $$\begin{aligned} \tilde{q}_{12}&= \tilde{F}_{12} \frac{c}{\tilde{H}_{12} + c}, \end{aligned}$$

75 $$\begin{aligned} \tilde{q}_{21}&= \frac{ \left( k_{13} c - {k_{23}} \right) ^2}{ k_{13} k_{31} c + k_{23} k_{32}} \frac{k_{31}k_{32}}{k_{31} + k_{32}} = \tilde{F}_{21} \frac{\left( c - \frac{k_{23}}{k_{13}}\right) ^2}{\tilde{H}_{21} + c} \end{aligned}$$

76 $$\begin{aligned} \tilde{q}_{23}&= \frac{k_{13} k_{31} c + k_{23} k_{32}}{ k_{31} + k_{32} } = \tilde{k}_{23} c + \tilde{K}_{23}\end{aligned}$$

77 $$\begin{aligned} \tilde{q}_{32}&= k_{31} + k_{32} \end{aligned}$$

with

78 $$\begin{aligned} \tilde{F}_{12}&= \frac{k_{23}(k_{31} + k_{32})}{k_{31}}, \tilde{H}_{12} = \frac{k_{23} k_{32}}{k_{13} k_{31}} \end{aligned}$$

79 $$\begin{aligned} \tilde{F}_{21}&=\frac{k_{13} k_{32}}{ k_{31} + k_{32}} , \tilde{H}_{21} = \tilde{H}_{12} = \frac{k_{23} k_{32}}{k_{13} k_{31}} \end{aligned}$$

80 $$\begin{aligned} \tilde{k}_{23}&=\frac{k_{13} k_{31}}{ k_{31} + k_{32}} , \tilde{K}_{23} = \frac{k_{23} k_{32}}{k_{31} + k_{32}} \end{aligned}$$

For a  model with one activating binding site and one ligand-independent, we find that $\tilde{q}_{12}$ has the form of a Michaelis-Menten term with maximum rate $\tilde{F}_{12}$ and half-saturation constant $\tilde{H}_{12}$. For $\tilde{q}_{21}$ we obtain another Hill function type term with a numerator of degree 2 and denominator of degree 1. This means that for large *c*, rate $\tilde{q}_{21}$ behaves like a mass action term, although, initially, it even decreases until vanishing at $\frac{k_{23}}{k_{13}}$. The rate $\tilde{q}_{23}$ is a mass action rate boosted with a constant influx $\tilde{K}_{23}$. Thus, for a  model with one activating and one ligand-independent transition to the open state, a qualitatively different model is obtained. Rather than linearly increasing with the ligand concentration *c*, the transition rate $\tilde{q}_{12}$ shows a saturating behaviour—it tends to the maximum rate $\tilde{F}_{12}$ for large *c*. The rate $\tilde{q}_{21}$ is again ligand-dependent with a more complicated dependency on the ligand concentration *c* which, however, behaves like a mass action term for large *c*. The transition $\tilde{q}_{23}$ is a mass action term, but with an additive ligand-independent constant $\tilde{K}_{23}$.

#### One activating and one inhibitory binding site

We now consider the case that one of the two binding site facilitates the transition from  to the open state  whereas the other one increases the rate to the closed state  i.e. the channel has one activating and one inhibitory binding site

81 $$\begin{aligned} q_{13} = k_{13} c, q_{23} = k_{23}, q_{31} = k_{31}, q_{32} = k_{32} c. \end{aligned}$$

Using (63)-(66), the rates of the model  are:

82 $$\begin{aligned} \tilde{q}_{12}&= \frac{k_{13} k_{23} (k_{31} + k_{32}c) }{k_{13} k_{31} + k_{23} k_{32}} = \tilde{k}_{12} c + \tilde{K}_{12}, \end{aligned}$$

83 $$\begin{aligned} \tilde{q}_{21}&= \frac{ \left( k_{13} c - {k_{23}} \right) ^2}{ k_{13} k_{31} + k_{23} k_{32}} \frac{k_{31}k_{32}}{k_{31} + k_{32} c} = \tilde{F}_{21} \frac{\left( c - \frac{k_{23}}{k_{13}}\right) ^2}{\tilde{H}_{21} + c} \end{aligned}$$

84 $$\begin{aligned} \tilde{q}_{23}&=\frac{k_{13} k_{31} + k_{23} k_{32}}{ k_{31} + k_{32} c } c= \tilde{F}_{23} \frac{c}{\tilde{H}_{23} + c} \end{aligned}$$

85 $$\begin{aligned} \tilde{q}_{32}&= k_{31} + k_{32} c = \tilde{k}_{32} c + \tilde{K}_{32} \end{aligned}$$

with

86 $$\begin{aligned} \tilde{k}_{12}&= \frac{k_{12} k_{23}k_{32}}{k_{13}k_{31} + k_{23}k_{32}}, \tilde{K}_{12} = \frac{k_{12} k_{23}k_{31}}{k_{13}k_{31} + k_{23}k_{32}}, \end{aligned}$$

87 $$\begin{aligned} \tilde{F}_{21}&=\frac{k_{13}^2 k_{31}}{ k_{13}k_{31} + k_{23} k_{32}} , \tilde{H}_{21}= \frac{k_{31}}{k_{32}} \end{aligned}$$

88 $$\begin{aligned} \tilde{F}_{23}&=\frac{k_{13} k_{31} + k_{23} k_{32}}{k_{32}} , \tilde{H}_{23} = \tilde{H}_{21}= \frac{k_{31}}{k_{32}}, \end{aligned}$$

89 $$\begin{aligned} \tilde{k}_{32}&= k_{32}, \tilde{K}_{32}=k_{31}. \end{aligned}$$

For a model with one activating and one inhibitory binding site, we see similar terms as for the model with one activating and one ligand-independent transition that we considered in the previous section. Here, $q_{12}$ and $q_{32}$ are mass action terms with an additional ligand-independent offset. The transition to the open state, $q_{23}$, is again represented by a Michaelis-Menten term whereas the rate $\tilde{q}_{21}$ tends to a mass action rate for large *c*.

#### Summary

Our analysis of different mechanisms of ligand binding in the model  has shown that by transformation to the model , the dependency of some rates on the ligand concentration *c* might be changed from mass action kinetics to a Michaelis-Menten term, see Sections 3.5.2 and 3.5.3. In addition, even if all rates in the transformed model  are mass action terms, an additional ligand dependency might appear (here, in the transition $q_{21}$ between the closed states  and , see Section 3.5.1) . It remains to be seen if these differences are sufficient to allow, in practice, to distinguish between independent binding sites (as represented in the model ) and sequential binding (as in the model ) by comparing fits of both model structures to experimental data.

## Discussion

We have given an overview of the identifiability theory of aggregated Markov models. Aggregated Markov models appear naturally in the modelling of ion channels which generate stochastic dynamics $\mathcal {D}$ that consists of alternating sequences  and  of open and closed times. Using aggregated Markov models, this dynamics is described by transitions between (usually multiple) open and closed states  and .

We have first explained how aggregated Markov models can be enumerated i.e. we have shown how the number of different aggregated Markov models for a given number  of states can be calculated using Pólya enumeration. The results, which can be found in Table 1, clearly show that the number of aggregated Markov models grows extremely rapidly—for only $n_{\text {V}}=10$ vertices, we have more than 10 billion models! If we are inclined to interpret each model as a representation of a different biophysical “process”, the results in Table 1 are somewhat reduced due to non-identifiability but nevertheless increases quickly with the number of vertices.

The phenomenon of non-identifiability appears somewhat counter-intuitive at first glance—how can a “biophysical process” that is represented by transitions between the open and closed states of a Markov model not be reconstructed from the sequence of open and closed times generated by this “process”? In order to gain a better understanding of this problem, let us consider an open time . Due to the presence of multiple open states, it is not clear, by which of the open states  the open time  has been generated—or if, during the time , the model has even alternated between multiple open states. This raises the question how much detail of the parameters and/or the structure of an aggregated Markov model can be inferred at all from the dynamics $\mathcal {D}$ i.e. a sequence of open and closed times.

To answer these questions, we have not related models directly to the dynamics $\mathcal {D}$. Instead, we have considered reparametrisations of the infinitesimal generator *Q* of a given aggregated Markov model. This relies on a classical result by Fredkin et al. (1985); Fredkin and Rice (1986) that the dynamics $\mathcal {D}$ of an aggregated Markov model with  open and  closed states is characterised by the bivariate distributions  and . This implies that reparametrising a model *Q* so that the distributions  and  remain unchanged will yield a model $\tilde{Q}$ whose dynamics $\mathcal {D}$ is indistinguishable from the original model *Q* i.e. *Q* is *not parameter-identifiable*.

If such a model *Q* which lacks parameter identifiability is used for modelling ion channels, it will represent the dynamics $\mathcal {D}$ as well as any reparametrised version $\tilde{Q}$ of *Q*. Thus, the choice of parameters $q_{ij}$ for representing the dynamics $\mathcal {D}$ is not unique—multiple different parameter set (as we have seen, in most cases, infinitely many) represent the dynamics equally well.

If our primary aim is to find a model that reproduces the dynamics $\mathcal {D}$ accurately, this might not be considered a very important problem. Indeed, phase type distributions (Neuts 1978)—which can be interpreted as sojourn time distributions  of an aggregated Markov model with only one open state—are popular generalisations of the exponential distribution for waiting times. Here, non-identifiability is not a concern because the only role of the states (“phases”) is to accurately describe a given data set, not to represent particular “states” of a system. But if our goal is to build aggregated Markov model that represent aspects of the underlying biophysics of the observed dynamics, not being able to unambiguously determine the rates $q_{ij}$ implies that a non-identifiable model does not allow us to gain insight into the transition rates between different biophysical states.

We have investigated the consequences of non-identifiability by considering the fully connected three-state model. For a given parameter set, there usually exists an infinite equivalence class of models. Under certain conditions, this ambiguity could be resolved by reducing the non-identifiable fully-connected model which depends on six rates to either of the parameter-identifiable models  or  which depend on four rates.

However, crucially, this model reduction does not allow us to *rule out* the presence of particular transitions between conformational states that are represented in the fully connected model. Rather, this only demonstrates that given dynamics $\mathcal {D}$ *can* be explained in the absence of some of these transitions between states of the channel protein. But going further, model reduction does not even allow us to distinguish between the models  and —as we have seen, if a parameter set of the fully connected three-state model can be reduced to the model structure  it can also be reduced to  and vice versa. And despite the fact that the parameters $q_{ij}$ are identifiable for either of the two models, the *model structure* $\mathcal {G}$ for models with  open and  closed states remains non-identifiable—it is not possible to decide if  or  provides a better representation of the dynamics $\mathcal {D}$. This shows that it is not possible to distinguish if the dynamics $\mathcal {D}$ is generated by one “fast” and one “slow” closed state from which the channel can transition to the open state—this mechanism is represented by the model . Or, alternatively, via a stepwise process where the channel makes a transition from a closed state  to a state  which primes the channel for entering the open state . The two mechanisms are, obviously, also very different from a biophysical point of view—if we assume, as in Section 3.5—that the transitions between states are regulated by binding sites, the model  represents a channel with two independent binding sites whereas  is based on the assumption of sequential binding.

Interestingly, reducing the non-identifiable fully connected three-state model to an identifiable model with four instead of six rates is not always possible because, for some parameter sets, reparametrising the model would lead to negative rates. In this situation, the non-identifiability cannot be resolved by choosing a particular model, simply because it is not obvious which model to pick as a representative for the infinite sets of equivalent models of the equivalence class. Interpreted from a biophysical point of view, in this case it is not possible to infer, which conformational changes might have generated the observed dynamics $\mathcal {D}$. In the case of the fully connected three-state models, this applied to models for which the sojourn time distribution  could not be represented as a mixture of exponential distributions but as a signed mixture of exponentials. This shows that some dynamics $\mathcal {D}$ that aggregated Markov models can generate, cannot be represented with an identifiable model, or, in other words, some dynamics $\mathcal {D}$, in principle, cannot be associated unambiguously with a biophysical mechanism.

In summary, two lessons can be learnt. First, for some parameter sets, the fully connected model can be reduced to the model structures  and . This means that the dynamics *can* be represented by assuming a simpler, acyclic mechanism. But this does not exclude that the dynamics could equally well be generated by a mechanism where transitions between all states are possible. Also, it is not possible to distinguish between the fundamentally different mechanisms  and . Second, the fully connected three-state model can generate sojourn time distributions based on a signed mixture of exponential whose densities show local maxima and minima rather than the monotonously decreasing densities obtained from a “normal” mixture of exponential distributions (where all coefficients of the exponentials are positive and form a finite probability distribution). These sojourn time distributions cannot be represented so that the model is parameter-identifiable so that these dynamics cannot be associated with transitions between different conformational states of the channel protein.

It has been suggested that the problem of identifiability of model structure (i.e. of the underlying biophysical mechanism) might be addressed by considering multiple data sets. We have investigated the question if the two alternative three-state models  and  could be distinguished when the dependency of a channel on the concentration *c* of a ligand is considered. Starting from models based on particular assumptions for different ligand binding sites, we have investigated how the ligand dependencies differ in the transformed model  after reparametrising the model . For some models, our results show that some transitions in the transformed model  are enzyme kinetics whereas in the original model , mass action was assumed. This does not help to distinguish  and  because alongside mass action kinetics, Michaelis-Menten terms (or other Hill functions) are common models of ligand binding in ion channel modelling. A clearer hint that dynamics $\mathcal {D}$ could be better described by the  model than the  model, might be the emergence of ligand-dependent transitions where they would normally not be expected—the most striking example is the case of the  model with two activating binding sites, see Section 3.5.1: here, the mass action rates $q_{13}(c)$ and $q_{23}(c)$ connecting the closed states  and  to the open state  in the model  translate to analogous rates $q_{12}(c)$ and $q_{23}(c)$ in the transition from closed state  via  to the open state  in the model . But the emergence of a mass action rate $q_{21}(c)$ in the transition from  to  is unexpected and would unlikely be chosen as such if the model had been developed based on mechanistic assumptions. It remains to be seen if criteria like this can be exploited in practice when comparing the fits of equivalent ion channel models to the same data set.

Having explored the non-identifiability of aggregated Markov models, we will reflect on implications for modelling ion channels and indicate how mechanistic data-driven models can nevertheless be developed under these circumstances. Beyond the sufficient condition of  for the maximum number of rate constants of an aggregated Markov model introduced by Fredkin et al. (1985); Fredkin and Rice (1986), to the best of our knowledge, no other criteria are available that can be applied easily to decide if a given Markov model is identifiable. If it is unknown if a given model is identifiable, indirect information can be gained by using approaches that investigate the uncertainties of parameters. We have suggested specifically that Markov chain Monte Carlo (MCMC) can be useful for deciding if a model could be non-identifiable (Siekmann et al. 2012). Alternatively, profile likelihood approaches have been applied to a wide range of models to assess identifiability (Bates and Watts 1988; Raue et al. 2009; Kreutz et al. 2012, 2013; Simpson and Maclaren 2023; Ciocanel et al. 2024; Plank and Simpson 2024) although, to the best of our knowledge, they have not been applied specifically to aggregated Markov models.

An approach that attempts to address both the lack of parameter identifiability as well as non-identifiability of model structure is the use of *canonical forms*. Canonical forms as those proposed by Bauer et al. (1987); Kienker (1989) and Larget (1998); Bruno et al. (2005); Flomenbom and Silbey (2006) are model structures that are on the one hand parameter identifiable and on the other hand, provide representatives for each of the different model structures that exist for a given number of open and closed states. Restricting the set of models to be considered to representatives of an equivalence classes of models makes the process of model selection more efficient because it prevents the modeller from comparing fits of equivalent models (such as the models  and  investigated in detail in this study) which, in theory, should show identical performance when fitted to the same data. Although canonical forms solve the statistical problems related to non-identifiability when it comes to fitting aggregated Markov models to data, modellers are restricted to the model structures defined by the canonical forms which constrains their freedom of representing biophysical mechanisms. A more severe problem is that some data sets might only be represented by physically unrealistic models, for example, the *manifest interconductance rank (MIR)* form proposed by Bruno et al. (2005) as well as the BKU form (Bauer et al. 1987; Bruno et al. 2005) might only be able to model some data sets if some rates are allowed to be negative, similar to the example presented in Section 3.4. Nevertheless, the MIR form by Bruno et al. (2005) allows for a refined representation of the equivalence classes of aggregated Markov models because it considers the “interconductance rank”  i.e. the rank of the transition matrices  and  which consist of the rates connecting open and closed states. In MIR form, the number of transitions between open and closed states is chosen to coincide with the rank of  and . The MIR form therefore depends on  parameters, consistent with the refined upper bound for the maximum number of parameters derived in Fredkin and Rice (1986).

It remains a challenging problem to design models that represent the observed dynamics $\mathcal {D}$ of an ion channel but also capture biophysical processes such as conformational changes. Designing models based on detailed knowledge of activating and inhibitory binding sites and other molecular properties of the ion channel leads to models with complex structures consisting of a large number of states and many parameters. Thus, this approach is fraught with problems related to non-identifiability, or, even more simply, over-parametrisation. Building models that mimic aspects of the molecular structure of ion channels is based on the hope that the parameters of these models will reflect the transition rates between biophysical states of the ion channel and thus provide insights into the time scales of conformational changes.

We would like to contrast this with alternative approaches that provide more direct insight into the time scales of conformational changes by rigorous statistical analysis of the dynamics $\mathcal {D}$. Modal gating is a phenomenon that describes ion channels switching between different dynamical patterns characterised by different levels of activity (i.e. for example different levels of open probability). These modes form a limited repertoire of dynamical patterns between which the ion channel alternates instantaneously. Thus, from a mathematical point of view, modal gating is an example of dynamics that exhibits two clearly different time scales. The “fast” dynamics is characterised by different modes that are associated with particular dynamical patterns. On the “slow” time scale the ion channel switches between these modes.

Modal gating has been investigated statistically by Ionescu et al. (2007); Siekmann et al. (2014). Both have developed methods which enable us to quantitatively infer the time scales of switching between modes as well as characterise the dynamics of individual modes. It is well-established that modes are manifestations of different conformational states in the dynamics of an ion channel, see, in particular, the study Chakrapani et al. (2011) as well as a wealth of additional references reviewed in the Discussion of Siekmann et al. (2014). Thus, statistical analysis of modal gating enables us to infer the dynamics of conformational changes of the ion channel by associating modes with different conformational states of the ion channel. The importance of modal gating for the modelling of ion channels is increasingly recognised—modal gating dynamics was included in Ullah et al. (2012) as one of multiple data sources. Siekmann et al. (2012) and Bicknell and Goodhill (2016) built models whose structures were designed based on modal gating as the underlying construction principle and Siekmann et al. (2016) proposed a structure specifically for representing modal gating as a hierarchical process—the hierarchical Markov model combines a Markov model that represents the switching between different modes with models for the dynamics of the individual modes to a model that accounts for both switching between modes as well as the dynamics within modes. Due to the close relationships of modes and conformational states, the states of the hierarchical Markov model can be interpreted to represent conformational changes, similar to models based on certain assumptions on biophysical processes such as ligand binding. But in contrast to these models, in the hierarchical Markov model, the rates between different conformational states can be parameterised based on a rigorous statistical analysis of the transitions between modes observed in the data.

Additional insight into both the dynamics as well as the molecular structure of ion channels can be gained from data that characterises the latency of an ion channel i.e. the delayed response of an ion channel to changes in ligand concentrations. Hawker et al. (2025) developed a method for extending an existing model whose ligand dependency was parametrised using multiple data sets at constant concentrations *c* by integrating additional data that investigated the response of the channel to changes of the ligand concentration. The biophysical significance of this model can best be understood by considering the transition from a ligand concentration $c_1$ to a ligand concentration $c_2$ where for concentration $c_1$ the channel protein is in conformation A with a high probability whereas for concentration $c_2$ the channel is most likely in different conformational state B. When instantaneously switching from $c_1$ to $c_2$ the channel cannot immediately adjust to the new ligand concentration, instead it transitions from A to B with a delay. This delay is represented in the model by introducing an integral term that averages the ligand concentration over a certain finite time interval. Thus, instead of an immediate switch the integral introduces a gradual transition from $c_1$ to $c_2$ and instead of an immediate “jump” the transition from conformation A to conformation B comes with a delay.

In summary, our study has demonstrated that non-identifiability poses difficult challenges for developing models that represent both the dynamics $\mathcal {D}$ as well as the biophysics of an ion channel by identifying individual states of a Markov model with conformational states of the channel protein. But we have presented some alternative approaches for developing models that represent conformational changes underlying the opening and closing of an ion channel by integrating additional data. By giving this detailed introduction into the complex phenomenon of non-identifiability we hope to facilitate the task of building mechanistic data-driven models of ion channels and, more general, taking advantage of the framework of aggregated Markov models for other applications.

## Data Availability

This study has no associated data.

## Footnotes

1 Although not the original developer of the theory, Pólya has been very influential in its popularisation and extension in the seminal article (Pólya 1937).

2 The application of Pólya enumeration to graphs is presented in the monograph by Harary and Palmer (1973).

3 In terms of integer sequences the relationship (11) is also known as an Euler transform.

4 More formally, the statement that “all information on the dynamics of an aggregated Markov model is contained in the bivariate distributions  and ” means that the parameters of higher-order distributions such as  or  are uniquely determined by the bivariate distributions  and . See Fredkin et al. (1985); Fredkin and Rice (1986).

5 Note that by considering the distributions  and , the upper bound for parameters derived by Fredkin et al. (1985); Fredkin and Rice (1986) is only valid for processes that are at equilibrium. Recordings from single ion channels are usually started after the channels had sufficient time to adjust to the experimental conditions such as, for example, ligand concentrations. For this reason, the assumption that the ion channel dynamics $\mathcal {D}$ has reached equilibrium is justified. A theory for the non-identifiability of aggregated Markov models that have not reached the stationary distribution has been developed by Ito et al. (1992) for discrete-time aggregated Markov models and was then transferred to the continuous case by Rydén (1996).

6 More than 10 years ago, Edmund Crampin and I had several passionate (but enjoyable) discussions about the “right” approach for modelling ion channels. Edmund suggested that I look at the relationship between different models for ligand binding and non-identifiability. This section is a first attempt to investigate the implications of non-identifiability on mechanistic models.
